# Signal transduction activated by the cancer chemopreventive isothiocyanates: cleavage of BID protein, tyrosine phosphorylation and activation of JNK

**DOI:** 10.1054/bjoc.2000.1636

**Published:** 2001-03

**Authors:** K Xu, P J Thornalley

**Affiliations:** Department of Biological Sciences, University of Essex, Central Campus, Wivenhoe Park, Colchester, Essex, CO4 3SQ, UK

**Keywords:** isothiocyanate, BID, JNK, tyrosine phosphorylation, apoptosis

## Abstract

Phenethyl isothiocyanate and allyl isothiocyanate induce apoptosis of human leukaemia HL60 cells in vitro. Apoptosis was associated with cleavage of p22 BID protein to p15, p13 and p11 fragments and activation of JNK and tyrosine phosphorylation (18 kDa and 45 kDa proteins). All these effects and apoptosis were prevented by exogenous glutathione (15 mM). Protein tyrosine phosphatase activity was unchanged. The general caspase inhibitor Z-VAD-fmk prevented apoptosis but not JNK activation – excluding a role for caspases in JNK activation, whereas curcumin prevented JNK activation but only delayed apoptosis. This suggests that in isothiocyanate-induced apoptosis, the caspase pathway has an essential role, the JNK pathway a supporting role, and inhibition of protein tyrosine phosphatases is not involved. © 2001 Cancer Research Campaign http://www.bjcancer.com


*British Journal of Cancer* (2001) **84**(5), 670–673
© 2001 Cancer Research Campaign
doi: 10.1054/ bjoc.2000.1636, available online at http://www.idealibrary.com on IDEAL
http://www.bjcancer.com

Short Communication

### Signal transduction activated by the cancer chemopreventive isothiocyanates: cleavage of BID protein, tyrosine phosphorylation and activation of JNK

**K Xu and PJ Thornalley**

Department of Biological Sciences, University of Essex, Central Campus, Wivenhoe Park, Colchester CO4 3SQ, Essex, UK

**Summary** Phenethyl isothiocyanate and allyl isothiocyanate induce apoptosis of human leukaemia HL60 cells in vitro. Apoptosis was associated with cleavage of p22 BID protein to p15, p13 and p11 fragments and activation of JNK and tyrosine phosphorylation (18 kDa and 45 kDa proteins). All these effects and apoptosis were prevented by exogenous glutathione (15 mM). Protein tyrosine phosphatase activity was unchanged. The general caspase inhibitor Z-VAD-fmk prevented apoptosis but not JNK activation – excluding a role for caspases in JNK activation, whereas curcumin prevented JNK activation but only delayed apoptosis. This suggests that in isothiocyanate-induced apoptosis, the caspase pathway has an essential role, the JNK pathway a supporting role, and inhibition of protein tyrosine phosphatases is not involved. © 2001 Cancer Research Campaign http://www.bjcancer.com

**Keywords**: isothiocyanate; BID; JNK; tyrosine phosphorylation; apoptosis

Dietary isothiocyanates such as phenethyl isothiocyanate (PEITC) and allyl isothiocyanate (AITC) have anti-carcinogenic activities and are of potential use in the chemoprevention of cancer (Hecht, 1995). Chemopreventive activity is associated with inhibition of the activation of carcinogens by cytochrome P450 isozymes (Conaway et al, 1996), increased excretion of carcinogens by quinone reductase and GSH S-transferases (Bogaards et al, 1990; Zheng et al, 1992), and also induction of apoptosis in pre-clinical tumours (Nishikawa et al, 1997; Samaha et al, 1997; Sugie et al, 1999). The mechanism of induction of apoptosis is unknown.

PEITC and AITC inhibited the growth and induced apoptosis of human leukaemia (HL60) cells in vitro (Adesida et al, 1996; Xu and Thornalley, 2000). PEITC-induced apoptosis was characterized by entry of PEITC into cells and rapid formation and expulsion of the glutathione adduct S-(N-phenethylthiocarbamoyl)glutathione (PETC-SG), and protein thiocarbamoylation – exacerbated by the decrease in cellular glutathione (Xu and Thornalley, 2001). This committed the cells to apoptosis with a critical activation of caspase-8 in the initial 3 h and later activation of caspase-3 (Xu and Thornalley, 2000). The specific caspase-8 inhibitor Z-IETD-fmk, the general caspase inhibitor Z-VAD-fmk and a high concentration of glutathione (15 mM) inhibited PEITC-induced apoptosis completely (Xu and Thornalley, 2001). PEITC was found to induce a sustained activation of JNK1 in HeLa cells (Yu et al, 1996) and Jurkat cells in vitro (Chen et al, 1998). This was associated with activation of MEKK1 (Chen et al, 1998). Overexpression of Bcl-2 and Bcl-$x_L$ suppressed both PEITC-induced activation of JNK1 and apoptosis, suggesting that Bcl-2 and Bcl-$x_L$ could intervene upstream of JNK1 activation in PEITC-induced apoptosis (Chen et al, 1998). Curcumin is an inhibitor of JNK signalling upstream of MEKK1 (Chen and Tan, 1998) and it delayed the induction of apoptosis by PEITC (Xu and Thornalley, 2001).

Recent investigations of caspase-8 activated apoptosis have suggested a critical role of cleavage of the cytosolic protein BID (Bossy-Wetzel and Green, 1999; Gross et al, 1999) and a role for caspases in the activation of MEKK1 in the JNK pathway (Cardone et al, 1997). Protein tyrosine phosphorylation and inhibition of protein tyrosine phosphatase activity has been associated with apoptosis where JNK activation was involved (Lumelsky and Schwartz, 1996; Chen et al, 1999). Potent thiol-modifying agents such as isothiocyanates may induce apoptosis by inhibiting protein tyrosine phosphatase activity (Denu and Tanner, 1998). Our recent work has indicated that peptide and protein thiol modification by isothiocyanates may play a critical role in activating apoptosis. We describe here, for the first time, experiments designed to examine these features of isothiocyanate-induced apoptosis.

### MATERIALS AND METHODS

### Chemicals

PEITC and AITC were purchased from Aldrich Chemical Co Ltd (Poole, UK). The caspase inhibitor N-benzyloxycarbonyl-Val-Ala-Asp(OMe)-fluoromethylketone (Z-VAD-FMK) was purchased from Calbiochem (Nottingham, UK). [$\gamma$-$^{33}$P]Adenosine 5′-triphosphate was purchased from NEN Life Science Products, Inc (Stevenage, UK). Mouse monoclonal anti-phosphotyrosine IgG antibody (clone PT66, ascites fluid), horseradish peroxidase (HRP)-conjugated goat anti-mouse IgG were purchased from Sigma Chemical Co Ltd (Poole, UK). Goat polyclonal anti-BID antibody and HRP-conjugated donkey anti-goat IgG were purchased from Santa Cruz Biotechnology Inc (Santa Cruz, USA). Streptavidin-HRP (SAv-HRP) conjugate was purchased from Pharmingen (San Diego, USA). Biotinylated SDS-PAGE molecular mass protein standards (6.5–200 kDa) were purchased from Bio-Rad Laboratories Ltd (Hemel Hempstead, UK). Nitrocellulose

*Received 20 June 2000*
*Revised 7 November 2000*
*Accepted 17 November 2000*

*Correspondence to:* PJ Thornalley membranes were purchased from Sartorius Ltd (Epsom, UK). Enhanced chemiluminescence (ECL) Western blotting reagents were purchased from Amersham Life Science (Amersham, UK). Protein tyrosine phosphatase (PTP) assay and JNK kinase assay kits were purchased from New England BioLabs (Hitchin, UK).

### Cell culture

HL60 cells were cultured in RPMI 1640 media containing 10% fetal calf serum under an atmosphere of 5% $CO_2$ in air, 100% humidity and 37˚C (Adesida et al, 1996). Cells were seeded at $5 \times 10^4$ ml$^{-1}$ and incubated with and without 5 or 10 µM PEITC or AITC, and with and without other agents (15 mM GSH, 50 µM curcumin or 50 µM Z-VAD-fmk).

### Sodium dodecyl sulphate-polyacrylamide gel-electrophoresis (SDS-PAGE)

Electrophoresis was performed with 12% (w/v) SDS-polyacrylamide separating gels (80 mm × 60 mm × 1 mm), similar to the method described (Allen et al, 1993). Electrophoresis was performed for 1.5 h at 20 mA constant current.

### Measurement of protein tyrosine phosphorylation

HL60 cells ($5 \times 10^5$ ml$^{-1}$, 20 ml) were incubated with and without isothiocyanates and other agents for the times indicated, washed twice with ice-cold phosphate-buffered saline (PBS) and the cell pellets lysed in 50 µl of lysis buffer (1% Nonidet P-40, 137 mM NaCl, 10% glycerol, 1 mM phenylmethylsulphonyl fluoride, aprotinin (0.15 U ml$^{-1}$), 1 mM $Na_3VO_4$ and 20 mM Tris-HCl, pH 8) for 30 min on ice. Lysates were centrifuged (15 000 ***g***, 20 min, 4˚C). Two-fold concentrated SDS-sample buffer (217 mM Tris-HCl, pH 6.7; 17.4% glycerol, 5% SDS, 9% 2-mercaptoethanol, 0.0017% bromophenol blue) was added to cell lysate, boiled for 5 min and electrophoresed on a 12% SDS-polyacrylamide gel at 20 mA for 2 h. Biotinylated molecular mass protein standards (6.5–200 kDa) were run concurrently. Proteins were transferred electrophoretically to nitrocellulose membranes (35 mA, 1 h). Membranes were blocked for 1 h at room temperature with 5% milk protein, 0.1% Tween 20 in PBS (PBS-Tween), rinsed twice followed by three 10 min washes with PBS-Tween. Membranes were probed at room temperature with monoclonal anti-phosphotyrosine IgG at 1:2000 dilution in PBS-Tween with 3% milk protein for 1 h. After washing, membranes were probed with HRP-conjugated goat anti-mouse IgG at 1:10 000 dilution in PBS-Tween with 3% milk protein for 1 h. Molecular mass standards were probed with SAv-HRP at 1:1000 dilution in PBS-Tween with 3% milk protein. After washing, blots were developed with the ECL detection system.

### Assay of protein tyrosine phosphatase

Protein tyrosine phosphatase (PTP) activity was assayed by measuring the rate of dephosphorylation of tyrosine [$^{33}$P]phosphorylated myelin basic protein (MyBP). [$^{33}$P]MyBP was prepared from MyBP with γ-[$^{33}$P]-ATP and Abl protein tyrosine kinase according to the manufacturer's instructions (New England BioLabs), giving a solution of 2.0 µM [$^{33}$P]MyBP with a tyrosine phosphorylation of 0.48. The PTP activity of HL60 cell lysates was determined by incubation of 30 µl of assay buffer (50 mM Tris-HCl, pH 7.5; 1 mM $Na_2$EDTA, 0.01% Brij 35, 1 mg ml$^{-1}$ bovine serum albumin), 10 µl of cell lysate, 30 µl of 2.0 µM [$^{33}$P]MyBP for 10 min at 30˚C. The reaction was terminated by addition of 200 µl of 20% TCA. The solution was placed on ice for 5–10 min, centrifuged (12 000 ***g***, 5 min, 4˚C) and 200 µl of supernatant removed, scintillation cocktail added and counted. The activity of PTP is given in units where one unit of PTP activity releases one nmol of phosphate from [$^{33}$P]MyBP per minute under assay conditions.

### Measurement of BID and BID fragmentation

The procedure was similar to the measurement of protein tyrosine phosphorylation, except that the primary antibody was goat polyclonal anti-BID antibody, and secondary antibody was HRP-conjugated donkey anti-goat IgG.

### Assay of JNK activity

This was performed according to the manufacturer's protocol (New England BioLabs). Briefly, after incubations, cell lysates were prepared, and incubated with c-Jun fusion protein beads overnight at 4˚C. After centrifugation (15 000 ***g***, 2 min, 4˚C), pellets were washed twice with 0.5 ml of lysis buffer (20 mM Tris-HCl, pH 7.4; 150 mM NaCl, 1 mM EDTA, 1 mM EGTA, 1% Triton, 2.5 mM sodium pyrophosphate, 1 mM β-glycerolphosphate, 1 mM $Na_3VO_4$, 1 µg ml$^{-1}$ leupeptin, 1 mM PMSF), and twice with 0.5 ml of kinase buffer (25 mM Tris-HCl, pH 7.5; 5 mM β-glycerolphosphate, 2 mM DTT, 0.1 mM $Na_3VO_4$, 10 mM $MgCl_2$). The pellet was re-suspended in 50 µl of kinase buffer supplemented with 100 µM ATP and incubated for 30 min at 30˚C. The reaction was terminated by addition of 50 µl of 2-fold concentrated SDS-PAGE sample buffer. Samples were boiled for 5 min, cooled and centrifuged (10 000 ***g***, 2 min, 4˚C), and loaded onto a 12% SDS-PAGE gel. SDS-PAGE, electrophoretic transfer to nitrocellulose with blocking by 2% (w/v) milk protein, and Western blotting with primary antibody (rabbit anti-phospho-c-Jun antibody, 1:1000 dilution) and secondary antibody (HRP-conjugated anti-rabbit antibody, 1:2000 dilution) were performed. Proteins were detected with the ECL detection system.

### RESULTS

The pro-apoptotic protein BID, when processed by caspase-8 and caspase-3 to the truncated form tBID, is a major initiator of mitochondrial dysfunction in apoptosis. When HL60 cell cytosolic extracts were blotted with anti-BID IgG, full-length BID protein of molecular mass 22 kDa was detected, with non-specific blotting of protein bands of >30 kDa (Figure 1A, lane 1). When HL60 cells were incubated with 10 µM PEITC for 6 h, cytosolic extracts indicated a loss of full-length BID and the appearance of fragments of molecular mass at 15, 13 and 11 kDa (Figure 1A, lane 2). This fragmentation was prevented by the addition of 15 mM GSH (Figure 1A, lanes 3 and 4).

PEITC and AITC activated JNK activity in HL60 cells. AITC was a stronger inducer of JNK activity than PEITC although AITC was slightly less cytotoxic (Xu and Thornalley, 2000) – the $TC_{50}$ values of PEITC and AITC were 4.95 µM and 11.0 µM, respectively. The induction of JNK activity was prevented by 15 mM GSH (Figure 2A). During PEITC-induced apoptosis, the activity of JNK was high after 3 h and decreased at 6 h and 9 h (Figure 2B).

© 2001 Cancer Research Campaign 

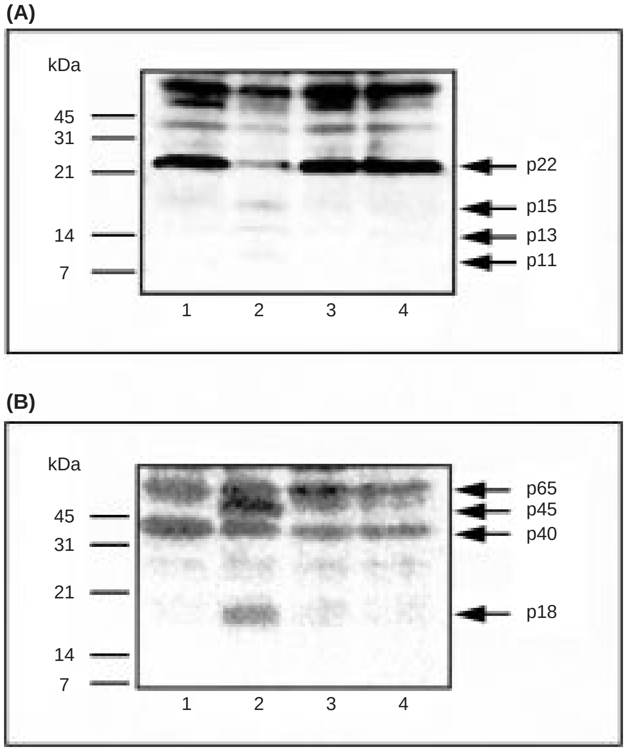

**Figure 1 (A)** Cleavage of cytosolic BID protein during PEITC-induced apoptosis of HL60 cells. BID protein (p22) and cleavage fragments (p11, p13 and p15) were detected by immunoblotting with anti-BID polyclonal antibody. Samples: cytosolic extracts of HL60 cells ($1 \times 10^7$) incubated for 6 h with and without additives at an initial cell density of $5 \times 10^5$ ml$^{-1}$. The blot shown was typical of 3 independent experiments. Key: lane 1, control; lane 2, PEITC (10 μM); lane 3, GSH (15 mM); and lane 4, PEITC (10 μM) + GSH (15 mM). (**B**) Tyrosine phosphorylation of 18 and 45 kDa proteins during PEITC-induced apoptosis HL60 cells. Phosphotyrosine residues were detected by immunoblotting with monoclonal anti-phosphotyrosine IgG antibody. Samples: cytosolic extracts of HL60 cells ($1 \times 10^7$) incubated for 6 h with and without additives at an initial cell density of $5 \times 10^5$ ml$^{-1}$. The blot shown was typical of 3 independent experiments. Key: lane 1, control; lane 2, PEITC (10 μM); lane 3, GSH (15 mM); and lane 4, PEITC (10 μM) + GSH (15 mM)

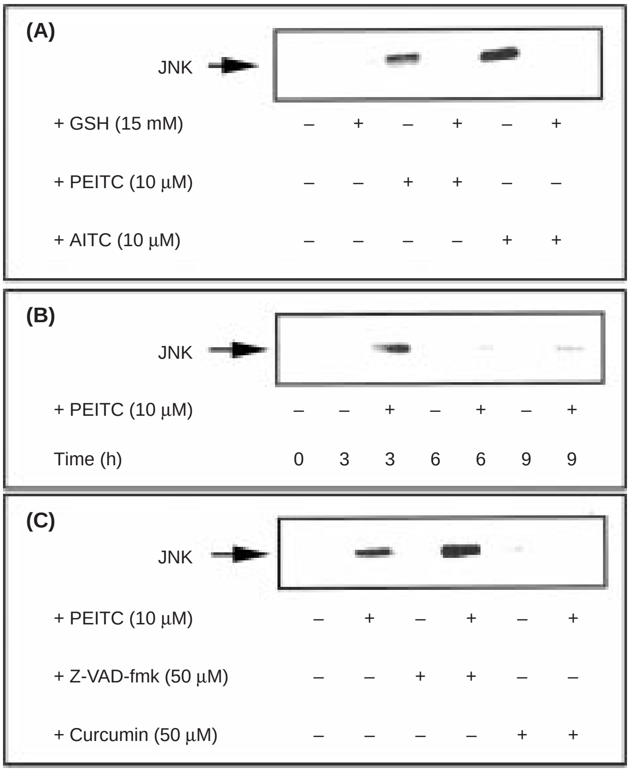

**Figure 2** Activation of JNK in HL60 cells during isothiocyanate-induced apoptosis and prevention by glutathione and curcumin but not by Z-VAD-fmk. HL60 cells ($5 \times 10^5$ ml$^{-1}$; $1 \times 10^7$) were incubated for 3 h with and without additives. Cell lysates were prepared, and JNK activity determined by a c-Jun fusion protein phosphorylation assay. Key: (**A**) Time course of JNK activation by PEITC, (**B**) prevention of PEITC and AITC-induced JNK activation by GSH, and (**C**) effect of Z-VAD-fmk and curcumin

The general caspase inhibitor Z-VAD-fmk (50 μM) did not inhibit the activation of JNK; in fact, it increased slightly the intensity of the blot, suggesting that it may have further increased JNK activity. Curcumin (50 μM), however, inhibited the activation of JNK (Figure 2C).

The tyrosine phosphorylation status of cytosolic proteins changed during PEITC-induced apoptosis was investigated. In cytosolic extracts of HL60 cells, two major protein bands of molecular mass ca. 40 kDa and 65 kDa were detected (Figure 1B, lane 1). When HL60 cells were incubated with 10 μM PEITC for 6 h, cytosolic extracts indicated the appearance of two new phosphoprotein bands of molecular mass 18 kDa and 45 kDa (Figure 1B, lane 2). This tyrosine phosphorylation was prevented by the addition of 15 mM GSH (Figure 1B, lanes 3 and 4).

Their formation was prevented by addition of 15 mM GSH. The effect of PEITC on the protein tyrosine phosphatase (PTP) activity of HL60 cells was studied. After 3 h, when the maximum binding of PEITC to cell protein occurred (Xu and Thornalley, 2001), the PTP activity was: control 2.12 ± 0.26, and + 10 μM PEITC 2.10 ± 0.11 ($n = 3$; $P > 0.05$).

### DISCUSSION

When HL60 cells were incubated with PEITC in vitro, caspase-8 and caspase-3 were activated (Xu and Thornalley, 2000). Caspase-8 and caspase-3 cleave BID protein to 3 fragments, p15, p13 and p11 fragments (Bossy-Wetzel and Green, 1999; Gross et al, 1999). This was found in PEITC-induced apoptosis of HL60 cells herein. p15 interacts with Bcl-$x_L$ in mitochondria, leading to cytochrome c release and loss of mitochondrial membrane potential (Li et al, 1998). A high concentration of GSH (15 mM) added to the extracellular medium prevented BID cleavage (this study) and apoptosis (Xu and Thornalley, 2001). Glutathione prevented the binding of PEITC to cells, probably by non-enzymatic formation of PETC-SG extracellularly. This keeps the PEITC concentration below cytotoxic levels. PETC-SG fragments to reform PEITC but there is a continuous slow hydrolysis of PEITC to inactive products that diminishes its pharmacological activity (Xu and Thornalley, 2000).

Both PEITC and AITC activated JNK in HL60 cells. JNK activation was mediated by MEKK1 (Yu et al, 1996; Chen et al, 1998). The strongest activation of JNK by PEITC occurred after 3 h when the concentration of cellular adducts of PEITC was maximal, the cellular GSH concentration was at a minimum and commitment to apoptosis occurred (Xu and Thornalley, 2000, 2001). Exogenous GSH prevented JNK but Z-VAD-fmk did not despite being an efficient inhibitor of caspase-3 and caspase-8 and, indeed, an efficient inhibitor of PEITC-induced apoptosis (Xu and

© 2001 Cancer Research Campaign

Thornalley, 2000). This excludes a role for caspases in the activation of MEKK1 (Cardone et al, 1997) in PEITC-induced apoptosis. The signalling upstream of MEKK1 is unknown. Curcumin suppressed the activation of JNK by PEITC and delayed but did not prevent the development of apoptosis (Xu and Thornalley, 2001). The role of JNK in apoptosis may be to potentiate cell death – JNK activation without executioner caspases did not induce apoptosis. JNK signalling increases the expression of fas ligand for increased agonism at the fas cell death receptor (Faris et al, 1998) and counters the anti-apoptotic activity of Bcl-$x_L$ in mitochondria (Kharbanda et al, 2000).

A critical role for tyrosine phosphorylation in signal transduction in apoptosis has been proposed (Chen et al, 1999), including apoptosis of HL60 cells (Lumelsky and Schwartz, 1996). Inhibition of protein tyrosine phosphatases has been shown to potentiate apoptosis (Chen et al, 1999) although the fas-associated protein tyrosine phosphatase (FAP-1) that is influential in countering fas-mediated apoptosis (Li et al, 2000) was not highly expressed in HL60 cells (Komada et al, 1997). Protein tyrosine phosphorylation was an early event in PEITC-induced apoptosis of HL60 cells. Protein tyrosine phosphatases may be susceptible to inhibition by isothiocyanates by modification of their active site cysteinyl thiol (Denu and Tanner, 1998). We were unable, however, to demonstrate an effect of PEITC on protein tyrosine phosphatases. Hence, increased protein tyrosine kinase activity is implicated in the increased protein tyrosine phosphorylation in isothiocyanate-induced apoptosis.

PEITC is in phase I clinical trial for the chemoprevention of cancer. It may eventually find use in the prevention of primary and secondary tumours in vivo. The induction of tumour apoptosis contributes to these chemopreventive effects (Nishikawa et al, 1997; Samaha et al, 1997; Sugie et al, 1999).

### ACKNOWLEDGEMENTS

This work was supported by the Ministry of Agriculture, Fisheries and Food (UK).

### REFERENCES

Adesida A, Edwards LG and Thornalley PJ (1996) Inhibition of human leukaemia 60 cell growth by mercapturic acid metabolites of phenethyl isothiocyanate. *Food Chem Toxicol* **34**: 385–392

Allen RE, Lo TWC and Thornalley PJ (1993) A simplified method for the purification of human red blood cell glyoxalase I. Characteristics, immunoblotting and inhibitor studies. *J Prot Chem* **12**: 111–119

Bogaards JJP, Ommen B, Falke HE, Willems MI and Van Bladeren PJ (1990) Glutathione S-transferase subunit induction patterns of brussel sprouts, allyl isothiocyanate and goitrin in rat liver and small intestinal mucosa. *Food Chem Toxicol* **28**: 81–88

Bossy-Wetzel E and Green DR (1999) Caspases induce cytochrome c release from mitochondria by activating cytosolic factors. *J Biol Chem* **274**: 17484–17490

Cardone MH, Salvesen GS, Widmann C, Johnson G and Frisch SM (1997) The regulation of anoikis: MEKK-1 activation requires cleavage by caspases. *Cell* **90**: 315–323

Chen Y-R and Tan T-H (1998) Inhibition of the c-Jun N-terminal kinase (JNK) signaling pathway by curcumin. *Oncogene* **17**: 178

Chen Y-R, Wang W, Kong T and Tan T-H (1998) Molecular mechanism of c-Jun N-terminal kinase-mediated apoptosis induced by anticarcinogenic isothiocyanates. *J Biol Chem* **273**: 1769–1775

Chen Y-R, Wang X, Templeton D, Davies RJ and Tan T-H (1999) The role of c-Jun N-terminal kinase (JNK) in apoptosis induced by ultraviolet C and γ radiation. *J Biol Chem* **271**: 31929–31936

Conaway CC, Jiao D and Chung F-L (1996) Inhibition of rat liver cytochrome P450 isozymes by isothiocyanates and their conjugates: A structure-activity relationship study. *Carcinogenesis* **16**: 2423–2427

Denu JM and Tanner KG (1998) Specific and reversible inactivation of protein tyrosine phosphatases by hydrogen peroxide: evidence for a sulfenic acid intermediate and implications for redox regulation. *Biochemistry* **37**: 5633–5642

Faris M, Kokot N, Latinis K, Kasibhatla S, Green DR, Koretzky GA and Nel A (1998) The c-jun N-terminal kinase cascade plays a role in stress-induced apoptosis in Jurkat cells by up-regulating fas ligand expression. *J Immunol* **160**: 134–144

Gross A, Yin X-M, Wang K, Wei MC, Jockel J, Milliman C, Erdjument-Bromage H, Temps P and Korsmeyer SJ (1999) Caspase cleaved BID targets mitochondria and is required for cytochrome c release, while BCL-$X_L$ prevents this release but not tumour necrosis factor-R1/Fas death. *J Biol Chem* **274**: 1156–1163

Hecht SS (1995) Chemoprevention by isothiocyanates. *J Cell Biochem* (suppl) **22**: 195–209

Kharbanda S, Saxema S, Yoshida K, Pandey P, Kaneki M, Wang QZ, Cheng K, Chen YN, Campbell A, Sudha T, Yuan ZM, Narula J, Weichselbaum R and Kufe D (2000) Translocation of SAPK/JNK to mitochondria and interaction with Bcl-$x_L$ in response to DNA damage. *J Biol Chem* **275**: 322–327

Komada Y, Inaba H, Zhou Y-W, Zhang X-L, Tanaka S, Azuma E and Sakurai M (1997) mRNA expression of fas receptor (CD95)-associated proteins (Fas-associated phosphatase-1/FAP-1, fas-associating protein death domain/FADD, and receptor-interacting proteins/RIP) in human leukaemia/lymphoma cell lines. *Brit J Haematol* **99**: 325–330

Li H, Zhu H, Xu C-J and Yuan J (1998) Cleavage of BID by caspase 8 mediates the mitochondrial damage in the Fas pathway of apoptosis. *Cell* **94**: 491–501

Li Y, Kanki H, Hachiya T, Ohyama T, Irie S, Tang G, Mukai J and Sato TA (2000) Negative regulation of fas-mediated apoptosis by FAP-1 in human cancer cells. *Internat J Cancer* **87**: 473–479

Lumelsky NL and Schwartz BS (1996) Protease inhibitors induce specific changes in protein tyrosine phosphorylation that correlate with inhibition of apoptosis in myeloid cells. *Cancer Res* **56**: 3909–3914

Nishikawa A, Lee I-S, Uneyama C, Furukawa F, Kim H-C, Kasahara K, Huh N and Takahashi M (1997) Mechanistic insights into chemopreventive effects of phenethyl isothiocyanate in N-nitrosobis(2-oxopropyl)amine-treated hamsters. *Jpn J Cancer Res* **88**: 1137–1142

Samaha HS, Kelloff GJ, Steele V, Rao CV and Reddy BS (1997) Modulation of apoptosis by sulindac, curcumin, phenylethyl-3-methylcaffeate and 6-phenylhexyl isothiocyanate: apoptotic index as a biomarker in colon cancer chemoprevention and promotion. *Cancer Res* **57**: 1301–1305

Sugie S, Yoshimi N, Okumara A, Tanaka T and Mori H (1999) Modifying effects of benzyl isothiocyanate and benzyl isocyanate on DNA synthesis in primary culture of rat hepatocytes. *Carcinogenesis* **14**: 281–283

Xu K and Thornalley PJ (2000) Studies on the mechanism of the inhibition of human leukaemia cell growth by dietary isothiocyanates and their cysteine adducts *in vitro. Biochem Pharmacol* **60**: 221–231

Xu K and Thornalley, P. J. (2001) Involvement of GSH metabolism in the cytotoxicity of the phenethyl isothiocyanate and its cysteine conjugate to human leukaemia cells *in vitro. Biochem. Pharmacol*, **61**: 165–177

Yu R, Jiao J-J, Duh J-L, Tan T-H and Kong A-NT (1996) Phenethyl isothiocyanate, a natural chemopreventive agent, activates c-Jun N-terminal kinase-1. *Cancer Research* **56**: 2954–2959

Zheng G, Kenney PM and Lam LKT (1992) Phenylalkyl isothiocyanates-cysteine conjugates as glutathione *S*-transferase stimulating agents. *J Med Chem* **35**: 185–188

© 2001 Cancer Research Campaign 

